# Does what we write matter? Determining the features of high- and low-quality summative written comments of students on the internal medicine clerkship using pile-sort and consensus analysis: a mixed-methods study

**DOI:** 10.1186/s12909-016-0660-y

**Published:** 2016-05-13

**Authors:** Lauren Gulbas, William Guerin, Hilary F. Ryder

**Affiliations:** School of Social Work, The University of Texas, Austin, TX USA; Geisel School of Medicine at Dartmouth, Hanover, NH USA; Department of Medicine, Dartmouth-Hitchcock Medical Center, One Medical Center Drive, Lebanon, NH 03784 USA

**Keywords:** Written comments, Cultural consensus, Medical education, Clinical medical education

## Abstract

**Background:**

Written comments by medical student supervisors provide written foundation for grade narratives and deans’ letters and play an important role in student’s professional development. Written comments are widely used but little has been published about the quality of written comments. We hypothesized that medical students share an understanding of qualities inherent to a high-quality and a low-quality narrative comment and we aimed to determine the features that define high- and low-quality comments.

**Methods:**

Using the well-established anthropological pile-sort method, medical students sorted written comments into ‘helpful’ and ‘unhelpful’ piles, then were interviewed to determine how they evaluated comments. We used multidimensional scaling and cluster analysis to analyze data, revealing how written comments were sorted across student participants. We calculated the degree of shared knowledge to determine the level of internal validity in the data. We transcribed and coded data elicited during the structured interview to contextualize the student’s answers. Length of comment was compared using one-way analysis of variance; valence and frequency comments were thought of as helpful were analyzed by chi-square.

**Results:**

Analysis of written comments revealed four distinct clusters. Cluster A comments reinforced good behaviors or gave constructive criticism for how changes could be made. Cluster B comments exhorted students to continue non-specific behaviors already exhibited. Cluster C comments used grading rubric terms without giving student-specific examples. Cluster D comments used sentence fragments lacking verbs and punctuation. Student data exhibited a strong fit to the consensus model, demonstrating that medical students share a robust model of attributes of helpful and unhelpful comments. There was no correlation between valence of comment and perceived helpfulness.

**Conclusions:**

Students find comments demonstrating knowledge of the student and providing specific examples of appropriate behavior to be reinforced or inappropriate behavior to be eliminated helpful, and comments that are non-actionable and non-specific to be least helpful. Our research and analysis allow us to make recommendations helpful for faculty development around written feedback.

## Background

In clinical medical education, written comments by a supervisor of a medical student provide documentation of student performance, demonstrate areas of academic strengths and weaknesses, provide the written foundation for student’s grade narratives and deans’ letters [1], and, as summative feedback, play an important role in the student’s professional development [2–5]. Written comments are a central element of medical education; a large body of research provides clear evidence that the characteristics of written feedback are important for its impact on learning [6]. Written comments are widely used and disseminated, yet, unlike Likert scale evaluations, little has been published about the quality of written comments.

A small body of literature exists on the subject of written comments about clinical learners in medical education. This literature focuses largely on the meaning of written comments. Research into the meaning of written comments about learners has used a single type of investigation - faculty analysis of the content of comments written by other faculty. These studies use written comments about third-year medical students [1, 3, 5, 7–9] or residents [6, 10–12] and code comments according to polarity (positive/negative) and internally-created subject categories, with category subjects varying from study to study and including such topics as “knowledge,” [10] “personal characteristics,” [1] “work ethic,” [9] “behavior,” [6] “future life as a physician,” [3] and “initiative” [7]. From this body of literature we learn that written comments are infrequently related to clinical skills [1] but often related to professional behaviors [7]. Overall, the majority of written comments have a positive polarity [3, 7, 9–11], but comments with negative polarity provide more discriminating information [3] and are viewed more seriously than those with positive or neutral polarity [12]. Faculty deem the quality of written comments given to students variable [1, 8, 11]. Analysis of written comment coding shows that faculty believe written comments are not specific enough to provide value to trainees [1, 6, 8].

A larger and related literature exists on the broader topic of giving feedback in medical education, much of it devoted to determining best practices [4, 13] and understanding conditions that enhance learner receptivity to feedback [14–16]. Lefroy et al. [13] note, “Helpful feedback… clarifies the trainee’s awareness of their developing competencies, enhances their self-efficacy for making progress, challenges them to set objectives for improvement, and facilitates their development of strategies to enable that improvement to occur.” If helpful feedback is crucial to the training of professional and competent medical students, we must be able to evaluate how medical students read and interpret feedback on their performance.

No published research has determined what students themselves think of written comments, and what they might find to be helpful. Indeed, research into whether students interpret comments in the same way is also lacking. Cultural consensus theory, from cognitive anthropology, can help us to determine whether students have shared beliefs around the quality of written comments. This theory offers a framework for estimating cultural beliefs and assumes that cultural beliefs are learned and held in common amongst a group [17]. As the amount of information in a culture is too large for any individual to master, individuals know different subsets of the common cultural knowledge or beliefs and vary in their cultural competence. Therefore, given a set of questions on a culturally relevant topic, shared cultural beliefs or norms regarding the answers can be estimated by aggregating the responses across a sample of culture members [18]. Cultural consensus models facilitate the discovery and description of possible consensus. Cultural consensus analysis has been widely used in medical settings, primarily to understand both patients' and physicians' cultural models [19–22].

We hypothesized that medical school is a culture shared by medical students. Specifically, medical students represent a professional cohort that carry specific attitudes, orientations, and beliefs about medical education, training, and evaluation. Given this, clinical medical students are likely to share an understanding, or cultural consensus, of qualities inherent to a high-quality and a low-quality written comment and that by using rigorous qualitative anthropological methods (described in detail below) we could successfully determine the features that clinical medical students use to define high and low quality comments.

## Methods

### Aim, participants, and setting

We aimed to show that medical students share an understanding of qualities inherent to high-quality and low-quality written comments and to determine features identifying high and low quality comments to clinical medical students. Because the primary aim of the study was to evaluate a shared culture among medical students, a purposive sampling strategy was used to select medical students in their third and fourth year of training. Sampling in this way ensured that participants had experience receiving evaluative feedback in written form. Recruitment for the study took place at an Ivy League Medical School in Northern New England, and the total sample included 22 students. For reliability at or above 0.90 in studies using cultural consensus analysis, samples between 20 and 30 individuals are recommended [24], and thus, our sample falls well within this range. More than half of participants in the sample were men (*n* = 13); 49 % of the classes sampled were male. The average age of participants was 26.3 years (range 22–33 years of age). Participants in our sample had experience reading and interpreting written comments as, at our institution, evaluations, including written comments, are available to the student upon completion of the clinical clerkship; clinical clerks at our institution are accustomed to reading written comments about their own performance and are accustomed to doing so out of the context of the clinical encounter. As with the majority of medical schools nation-wide, our school uses oral, formative feedback to improve the performance of the student over the course of the clerkship, and written, summative feedback is decontextualized and provided either in the form of grade narratives or raw evaluations weeks after the clinical experience.

### Design and data collection

Participants participated in a pile sorting activity to determine the helpfulness of each comment. Written comments were drawn from written assessments by supervising faculty clinicians of medical students two years prior (this time lag was to prevent any clinical medical student from evaluating a comment of their own performance).

After the research team read and reviewed all written comments, comments were segmented according to “meaning units,” or phrases or paragraphs that contain the same central meaning based on their content and context [23]. Meaning units were generated by the research assistant and reviewed and validated by the senior author. After segmenting comments, we randomly selected 62 segmented comments for inclusion in the pile sorting activity.

Pile sorting is a rigorous qualitative technique used within cognitive anthropology to examine how participants perceive items to be related [24]. Data collection for pile-sorting followed a two-step process outlined by Bernard and Ryan [25] and Weller [26]. First, participants were asked to sort items (written comments) into piles based on the perceived similarity. Specifically, participants were given cards, and each card contained one written comment. Participants were asked to sort cards into two piles: “unhelpful” and “helpful.” Thus, all written comments that were perceived to be similarly helpful were sorted in one pile, and comments that were perceived to be unhelpful were sorted into a different pile.

Second, participants were asked to describe, in their own words, their piles. Thus, following the sorting process, participants participated in a semi-structured qualitative interview to elicit their reasons for sorting each comment as helpful or unhelpful. Follow-up qualitative interviews are an essential component of collecting data when pile-sorting because descriptive answers (participant comments) obtained in the interview can be used to interpret the data gathered [25]. Open-ended questions were asked to understand what qualities contributed to a comment being perceived as helpful versus unhelpful, as well as perceived general characteristics of helpful and unhelpful comments. Each student provided participant comments on twenty randomly chosen written comments. We also performed a member-check with a small sample of participants to obtain their reflections about what was found and to shed more insight. This study was approved by the institutional review board of the medical school as well as the academic medical center.

### Data analysis

Data analysis proceeded in several stages to determine which written comments were perceived as helpful, and then, to determine why specific written comments were perceived as helpful. We performed cluster analysis first, and then analyzed the written comments in each cluster using both qualitative analysis and statistical analysis.

#### Cluster analysis

First, results from pile-sorts were analyzed using Visual Anthropac: Pile Sort [27]. Anthropac analyzes data along a given domain (in this case, the quality of written comments), and determines the degree of informant knowledge within a particular group. Specifically, Anthropac analyzes individual participant’s data, based on the percentage of participants who sorted any two items together in the same pile, to produce an aggregate similarity matrix that quantifies the percent of participants who sorted items (i.e., written comment) together in the same pile. Multidimensional scaling (MDS) is a non-metric means of visualizing the level of similarity of individual cases in a dataset and is also known as perceptual mapping. MDS obtains the underlying dimensions from respondents’ judgments about the similarity of two items and does not depend on researchers’ judgments. The underlying dimensions come from respondents’ judgments about pairs of items. MDS converts similarity data (such as the aggregate similarity matrix discussed above) in matrix form into a two-dimensional visual representation of the "distance" between sorted terms. Thus, using MDS analysis to analyze the aggregate similarity matrix, a data display was created to visually map in two-dimensional space how written comments were sorted similarly across all student participants [25]. The MDS map was then layered using the cluster analysis module to facilitate the identification of specific groups of items determined to be similar by students. In cluster analysis, items that share, on average, higher degrees of similarity are outlined visually into groups on the MDS map. Accordingly, cluster analysis permitted the research team to clearly demarcate written comments that were perceived as similar across the sample of participants.

Using the cultural consensus module in Anthropac, the degree of shared knowledge was calculated to determine the level of internal validity in the data [28]. The strength of the cultural consensus is evidenced by a value known as the eigenvalue, which serves as a goodness-of-fit indicator that a single factor (the cultural consensus of the group), is present in the pattern of responses. An eigenvalue of three or greater indicates that a group shares a common culture and consensus [29].

#### Qualitative analysis

In order to identify characteristics associated within helpful comments, data elicited during the semi-structured interview was transcribed and imported into Microsoft Excel for coding [30]. The coding process identified the specific words used by participants to describe how and why they evaluated written comments as helpful or unhelpful. The analysis of participant’s comments helped the research team to determine what contributed to the identification of a written comment as high-quality or low-quality. Participant’s comments were read, and the words they used to describe their evaluation of written comments were coded. When participants made explicit references to an example of a written comment, this information was also coded.

#### Statistical analysis

The analysis of participant’s comments was supplemented with statistical analysis to identify key patterns and characteristics associated with helpful written comments. Length of written comment was compared using one-way analysis of variance. Frequency with which comments were thought of as helpful were analyzed by chi-square. To determine the role of valence on the perceived quality of comments, the authors (HR and WG) performed independent structured coding of the data, grouping each comment into “positive,” “negative,” or “neutral” categories. Individual coding was compared and a consensus assignment given; kappa of >0.9 indicated high agreement. Valence within a cluster was treated as a categorical variable and analyzed by chi-square. In conducting statistical tests, our purpose was not to achieve statistical generalizability, but to systematically identify key differences *within* our sample.

## Results

Cluster analysis of written comments revealed four distinct clusters that varied significantly in both qualitative and quantitative statistical analysis.

Cluster A included 33 comments, perceived most frequently as “helpful” by clinical medical students. The subjects discussed in written comments in A spanned the gamut from strengths, recommendations for improvement, as well as comments on core competencies. These comments were noted by students to contain specific examples of the student’s behavior on the clerkship. Qualitative analysis of student discussion of these comments revealed that these comments were also thought by students to demonstrate knowledge of the student and relationship with the student. The comments either reinforced good behaviors or gave constructive criticism for how changes could be made and were found by students to contain information on how to excel in the student’s next clerkship. For example:

“Excellent communicator – compassionate and gifted in her communication with her patients, who were very fond of her and appreciative of her care; presentations and write-ups began as very competent and improved from there. Even the patients who were not ‘hers’ missed her when she left the rotation! Also excelled in communicating about ‘operational aspects,’ e.g. making sure team was aware of when she would be off to conference and when she would return.”

Two other clusters, Clusters B and C, were found to be less helpful by students. Cluster B, found to be unhelpful by 62 % of students, encouraged students to “keep up the good work,” in essence exhorting students to continue non-specific behaviors they had exhibited during their clinical experience. For example:

“Continue to read about your pts and offer changes to their mgmt. plans. You’re on the right start and it was a pleasure working w/you!”

Cluster C was found to be unhelpful by 51 % of students, and these comments described the student using terms found in the grading rubric of the medical school, without giving student-specific examples or advice on how to improve. For example:

“Enthusiastic, bright, energetic, excellent interpersonal skills with patients, team members, nursing, eager to learn, and ready to work.”

The last cluster, Cluster D, contained 18 comments and was found to be unhelpful by 87 % of students. Although the subject of comments in D also noted student strengths, provided recommendations for improvement, and commented on the core competencies; the traits shared by this group included use of third-person language (i.e., “He took good histories…”), sentence fragments lacking verbs and appropriate punctuation (i.e., “is above the level of his peers”, “was always punctual and prepared.”, “Be more confident in her plan.”). Overall, comments grouped into A were rated as helpful by significantly more students and cluster D comments were rated as helpful by significantly fewer students.

In general, when asked to define the characteristics of a helpful comment at the end of the pile-sort activity, 82 % of students used the word “specific” to define a helpful comment, and 45 % of students requested a concrete example or anecdote to illustrate the evaluator’s point:

“I want to see specifics – specific details in some situations and specific hints on improvement.”

“The comments I found most helpful were those that were specific to the student and brought in examples of the writer’s time with the student.”

When asked to define the characteristics of an unhelpful comment at the end of the pile-sort activity, 68 % of students used the words “generic”, “vague” or “non-specific” to define a non-helpful comment. With one student noting,

“In general, the comments I found unhelpful were vague – there was nothing the student could take away, no examples of how the student was doing well.”

### Statistical analysis

A one-way analysis of variance (ANOVA) was calculated on the length of each written comment and revealed significant differences between the groups, *F*(3, 57) = 14.73, *p* < 0.001. Comments in cluster A were longer than comments clustered into other groupings, with an average of 306 characters for A (*SD* = 153.2), versus 107 for B (*SD =* 22.9), 151 for C (*SD =* 46.7) and 91 for D (*SD =* 48.2) (see Table 1). A Chi-square test of independence was calculated comparing the proportion of students who deemed comments in each cluster to be helpful. Clusters varied significantly in whether the comments within each cluster were sorted to the helpful pile by students (*χ*^2^ (3) = 97.75, *p* < 0.001). The proportion of students sorting comments in cluster A as helpful was 0.81; the proportion of students sorting comments in cluster D as helpful was only 0.13. There was no difference between clusters B and C in terms of the proportion of students who sorted those comments as helpful (Cluster B 0.37 sorted as helpful; Cluster C 0.49 sorted as helpful).

**Table 1:** Cluster analysis of faculty written comments about students

Cluster	Number of comments	Average length of comment (SD) in characters	Proportion of students sorting comments in cluster as helpful	Features of cluster comments	Sample narrative comments	Student analysis of comments
A	33	306.4 (153.2)	0.81	Gives examples from student's clerkship; demonstrates knowledge of student	At her level of experience with hospital medicine, she demonstrated a solid understanding of the complex pathophysiology of common and uncommon diseases. I enjoyed her approach to patient care, which was well rounded and included psycho-social aspects as well as health-related aspects.	Shows close relationship between student and faculty and time put into understanding specific traits of the student.
				Helps student understand how to excel in next clerkship; reinforces good behaviors or gives constructive criticism for how to change	He would benefit from focusing on efficiency and being more assertive in putting forth his opinion on management decisions as he often has correct ideas and plans but hesitates to voice them.	1) Very specific about point of improvement, what is lacking and what needs focus. 2) Specific to the issue of hesitation and this goes a long way to instill confidence – something specific to take away.
B	3	107.3 (22.9)	0.37	Exhorts the student to continue current performance	Keep up the good work and speak up more on rounds and share your knowledge and thoughts about your patients.	N/A
C	7	151.1 (46.7)	0.49	Describes student using terms found in grading rubric without giving advice or specific information	Has a good fund of medication knowledge and demonstrates that she continues to read about patient presentation and pathology on a daily basis.	1) I don’t know what “good fund of knowledge” means. 2) The comment suggests they didn’t care enough to write a more helpful comment or simply didn’t know the student.
					Outstanding ability to synthesize and incorporate new knowledge, ideas, and organization into her thinking and proposed management for patients.	1) Vague. 2) I had no idea what this was even saying. What does it mean about exactly what her strengths were?
D	22	90.8 (48.2)	0.13	Use of third person without any personal descriptors or names	highly professional in all aspects of her conduct	Professionalism needs more specific details. A sentence like this is essentially useless – it doesn’t help the student or go in the Dean’s letter.
				Sentence fragments lacking verbs and capitalization	doesn’t have any specific deficiencies. He will benefit, as all of us do, from continuing to read and learn about each patient he sees	N/A
				No specific information given - often vague	is above the level of his peers. he did a great job on the short week I had with him.	1) This doesn’t tell me much – what is the level of my peers? 2) This is meaningless; the evaluator qualified ‘short week,’ really saying I didn’t know this student very well.

Chi-square 97.75, *p* < 0.001

F(3,57) = 14.73, *p* 0.001

### Role of valence on helpfulness

The majority of comments (61 %, 38/62) had a positive valence, while 29 % (18/62) had a neutral valance and 10 % (6/62) were negative. Valences between clusters did not vary significantly (*χ*^2^ (6) = 8.71, *p* = 0.19 (see Table 2). Sixty percent of the comments in both the most helpful cluster (Cluster A) and the least helpful cluster (Cluster D) had a positive valence. For example, Cluster A included this negative comment, found helpful by 91 % of students:

**Table 2:** Valence of clusters

		Valence of comment		
		Positive	Neutral	Negative
Number of comments in cluster	Cluster A	20	8	5
	Cluster B	1	2	0
	Cluster C	5	0	1
	Cluster D	12	8	0

Chi-square = 8.71, *p* = 0.19

“His history taking skills need a lot of improvement. He did not seem to be able to direct patients through the history taking process and took a very long time to complete his work. He is at the recorder stage which is understandable but he does not take initiative to look up the meaning of his recorded data before deferring to the team, which he should if he wants to build his fund of knowledge and learn good habits.”

Conversely, only 10 % of students found this Cluster D comment with positive valence helpful, “met and exceeded expectations for a new 3^rd^ year medical student with regards to medical knowledge.”

In all clusters, the cluster-related characteristics dominated valence – within a cluster, comments with negative valences shared the same characteristics as comments with positive valences.

### Shared model of comments

Eigenvalue ratio (a measure of the strength of group consensus with three or greater demonstrating very strong cultural consensus) was 12.1 for unhelpful comments and 10.0 for helpful comments, indicating strong cultural consensus among clinical medical students without evidence of subcultural variation. Stress (a measure of the goodness of fit of the data to the multidimensional scaling graph with <0.15 being acceptable and <0.1 being low) was 0.002 for unhelpful comments and 0.006 for helpful comments, indicating a good fit of the data to the model. In other words, student data exhibited a strong fit to the consensus model, supporting the assertion that, despite individual differences, all respondents in the sample belong to a single culture, or share a cultural model, of the features that make a written comment helpful or unhelpful.

## Discussion

This study is the first to explore how students interpret written comments about their clinical ward performance. Our research used well-established anthropological qualitative methods and applied them in a novel way to the field of clinical medical education. Through rigorous qualitative analysis, we determined that clinical medical students at our institution share a robust model or cultural consensus of the attributes of a helpful comment or unhelpful comment. Comments deemed to be most helpful by students included longer comments demonstrating knowledge of the student and comments providing specific examples of appropriate behavior to be reinforced or inappropriate behavior to be eliminated. These comments were specific and actionable. Universally, students found comments that were grammatically incorrect or lacked punctuation to be least helpful, along with comments that provided no student-specific information. In explaining their analysis, students stated that high-quality comments seemed to be written by faculty who knew their students. Feedback is defined as “specific information about the comparison between a trainee’s observed performance and a standard, given with the intent to improve trainee’s performance” [31] – it follows that a supervisor must know, and demonstrate knowledge, of both the trainee and a standard, to provide quality feedback such that it assists the trainee in improving their performance.

Our study also provides new evidence from the student’s perspective that credible evaluators providing specific information in the form of written comments or summative feedback can be perceived as helpful, even if the valence of the information is equivocal or negative. In our study, 61 % of the comments had positive valence. This is slightly lower than other published studies in which 70–94 % of analyzed comments were coded as positive [6, 7, 9–11, 32, 33]. The lower rate of positive comments is in line with other studies of faculty written comments to third-year clerkship students [9]; faculty written comments to residents [6, 10, 11] and to peers tend to be more markedly positive [33]. The perception that a comment was helpful was not associated with the valence of the comment – Cluster A, the highly helpful comments, had the same percent of positive comments as Cluster D, the least highly helpful comments. Prior research has suggested that complimentary remarks lead to greater student satisfaction than effective feedback [14]. We found, in our non-contextual exercise, that students *were* able to differentiate between what they might want to hear and what they might need to hear. Thus, our study also provides new evidence from the student’s perspective that credible evaluators providing specific information in the form of written comments or summative feedback can be received as helpful, even if the valence of the information is equivocal or negative.

Our study was subject to a few notable limitations. We enrolled clinical medical students from a single site and so generalizing our results to other levels of training (for example, graduate medical education) or other sites should be done with caution. Our sample size, approximately 15 % of the eligible students, was small, although data analysis did reach statistical significance. Students were asked to evaluate whether a written comment was helpful or not out of context; it could be that the threshold for helpfulness is different given appropriate context. In addition, our study was process oriented and not designed to determine whether comments perceived as helpful or unhelpful would have achieved a desired outcome of all feedback – influencing trainee behavior or improving clinical performance. Finally, not every student commented on their process of determining helpfulness for every comment. This may have led to incomplete understanding in our qualitative analysis as to why comments were clustered as they were.

## Conclusion

Our findings demonstrate that medical students share an understanding of the features or content of a helpful or unhelpful comment. Wide variation in the quality of comment was present at our academic tertiary care institution, and is present at many institutions [34]. Low-quality written feedback may be due to lack of training in providing effective feedback or poor feedback role modeling [6]. However, Holomboe et al. [35] find that faculty development modestly improves the quality of written feedback to residents. Creating awareness of the elements of helpful feedback may lead to improved written feedback on the part of supervising clinicians [6]. Several specific, student-centered recommendations to guide faculty development around written feedback may be made based on our research. First, faculty should be made aware that students thoughtfully and critically evaluate the quality and meaning of written evaluations. Second, students respond positively to written comments that indicate personal knowledge of the student and comments that provide specific examples of behaviors to reinforce or eliminate, and students seek these comments from their faculty. Third, while millennial students may often use casual, agrammatical, non-punctuated language in their social media and informal interactions, they prefer written comments to be written in formal, grammatically correct, appropriately punctuated and capitalized sentences. Further investigation should determine whether faculty members responsible for writing these comments share the same cultural consensus that we have noted within the medical school.

### Ethics approval

This study was performed in accordance with the Declaration of Helsinki and was approved by the institutional review board of the academic medical center, The Committee for the Protection of Human Subjects at Dartmouth College, study number STUDY00028120.

### Consent to participate

Informed consent to participate in the study was obtained from all participants.

### Consent for publication

Not applicable.

### Availability of data and materials

The dataset supporting the conclusions of this article is available upon request from the corresponding author.
